# The Role of Autoimmune Regulator (AIRE) in Peripheral Tolerance

**DOI:** 10.1155/2018/3930750

**Published:** 2018-09-04

**Authors:** Bingjie Zhao, Lu Chang, Haiying Fu, Guangyu Sun, Wei Yang

**Affiliations:** Department of Immunology, College of Basic Medical Sciences, Jilin University, Changchun 130021, China

## Abstract

Autoimmune regulator (AIRE), whose gene mutation is considered to be a causative factor of autoimmune polyglandular syndrome type 1 (APS1), is an important transcriptional regulator. Studies on the role of AIRE in the central immune system have demonstrated that AIRE can eliminate autoreactive T cells by regulating the expression of a series of tissue specific antigens promiscuously in medullary thymic epithelial cells (mTECs) and induce regulatory T cell (Treg) production to maintain central immune tolerance. However, the related research of AIRE in peripheral tolerance is few. In order to understand the current research progress on AIRE in peripheral tolerance, this review mainly focuses on the expression and distribution of AIRE in peripheral tissues and organs, and the role of AIRE in peripheral immune tolerance such as regulating Toll-like receptor (TLR) expression and the maturation status of antigen presenting cells (APCs), inducing T cell tolerance and differentiation. This review will show us that AIRE also plays an indispensable role in the periphery.

## 1. Introduction

Autoimmune regulator (AIRE) is an important transcriptional regulator that is mainly expressed in medullary thymic epithelial cells (mTECs) in the central immune system. AIRE can maintain central immune tolerance, since AIRE clears auto-reactive T cells and induces Treg production by regulating the expression of peripheral tissue-specific antigens (TSAs) in mTECs [1]. Mutations in AIRE cause autoimmune polyglandular syndrome type 1 (APS-1), also known as autoimmune polyendocrinopathy-candidiasis-ectodermal dystrophy (APECED). APS-1 is mediated by autoimmune responses and is characterized by multiple-organ injury; clinically, it appears as an autoimmune disease with polyglandular failure [2]. However, increasing evidence indicates that AIRE is also expressed in peripheral lymphoid organs and tissues. Stromal cells in the peripheral spleen and lymph node tissues, the embryonic liver, testis, and ovarian tissues, and haematogenic monocytes/macrophages and dendritic cells have all been shown to express AIRE [3]. Although the number of studies on the function of AIRE in peripheral tolerance has gradually increased, the roles and significance of AIRE in peripheral tolerance are not yet clear [4]. This study will review the AIRE protein structure, the distribution of peripheral AIRE expression, and the role of AIRE in peripheral immune tolerance.

## 2. Tissue and Cellular Distributions of AIRE

AIRE expression has been detected in tissues and cells from different locations in humans and mice. AIRE is mainly expressed in the thymus, and the expression of both RNA and protein has been detected. The expression is most prominent in mTECs [5, 6], although low levels of AIRE expression have been detected in myeloid dendritic cells (DCs) [7]. In addition, in situ hybridization and reverse-transcription polymerase chain reaction (RT-PCR) studies have detected AIRE mRNA expression in stromal cells in peripheral spleen and lymph node tissues and in embryonic liver, testis, and ovarian tissues [5]. Our study on the tissue and cellular distributions of peripheral AIRE showed that AIRE was expressed in peripheral immune organs (spleen and lymph nodes), reproductive organs (testis and ovary), bone marrow-induced DCs, peripheral blood mononuclear cells (PBMCs), and spleen DCs [8].

Regarding peripheral AIRE expression, the results of studies are not completely consistent. For instance, Halonen et al. detected strong AIRE expression in the lymph nodes and spleen of NRMI mice at both the RNA and protein levels; it was also detected in the bone marrow, peripheral blood cells, and ovaries [9]. Adamson et al. performed immunohistochemistry in inbred C57BL/6J and inbred CD1 mice and found strong protein staining in the spleen, lymph node, intestinal, gonad, and brain tissues and in epithelial cells in the lung, kidney, and oviduct, which was consistent with the RNA results [3]. Heino et al. reported the presence of AIRE mRNA in the thymus, lymph nodes, spleen, and other peripheral tissues in normal BALB/c mice; however, they did not detect AIRE protein expression in any tissue other than the thymus [7]. In humans, peripheral AIRE mRNA has been detected in the lymph nodes, tonsils, intestinal-associated lymphoid tissues, spleen, foetal liver, and PBMCs [10–13]. Heino et al. observed AIRE staining in the thymus medulla, paracortex zone, lymph nodes, spleen, and foetal liver medulla [12]. Poliani et al. also confirmed the presence of the AIRE protein in adult lymph nodes; however, the presence of the AIRE protein was not detected in many other peripheral tissues [10].

As mentioned above, the expression of AIRE in the periphery is still controversial. Given the differences of tissue origin and techniques used in these studies, there may be three aspects responsible for the inconsistent results. Firstly, if one considers the sensitivity of detection methods, taking the level of mRNA for instance, northern blot (NB) is unsuitable to detect AIRE mRNA even in the thymus. In contrast, researchers prefer RT-PCR or in situ hybridization (isH). In addition, in searching for the AIRE protein, when it comes to the accuracy of the detective Abs, greater accuracy comes from the use of monoclonal anti-AIRE Abs rather than polyclonal Abs. Last but not the least, the detection of AIRE mRNA in nonlymphoid organs remains questionable and could be due to the presence, in bulk tissue samples, of few AIRE-expressing cells (for example, elements of the monocyte/DC lineage) ordinarily inhabiting the organs or contaminating the preparations. In summary, it is best to use the monoclonal anti-AIRE Abs, especially if joined to other methods, such as flow cytometry, which can improve the purity of the cell samples [14].

At the cellular level, peripheral AIRE expression mainly occurs in one group of cells with a phenotype similar to DCs. Heino et al. showed that 10–20% of the AIRE-expressing cells in human lymph nodes expressed CD83, which was consistent with the DC phenotype [12]. Poliani et al. confirmed that these cells in human lymph nodes were CD45-negative DCs [10]; in addition, real-time polymerase chain reaction (PCR) showed that these cells expressed RNAs encoding indoleamine 2,3-dioxygenase (IDO) and interleukin- (IL-) 10 and that the expression of these molecules was consistent with the tolerance maintenance function [15]. Moreover, Suzuki et al. tested the AIRE expression in human peripheral B and T cells and CD14^+^ myeloid cells at the mRNA and protein levels. Based on the size and CD65 expression condition of these cells, Suzuki et al. classified these CD14^+^ cells as DCs/macrophages [13]. Zheng et al. compared the AIRE expression in CD11c^+^, CD11c^−^, and total splenocytes and used the enrichment and elimination of CD11c^+^ cells to detect changes in AIRE mRNA expression; their results confirmed that DCs were the major AIRE-expressing cells in the spleen [16]. Poliani et al. used double immunostaining to describe the properties of AIRE^+^ cells in human lymph nodes [10]. The results showed that AIRE^+^ cells always co-expressed HLA-DR and fascin and usually expressed the myeloid DC markers CD11c and S100. In addition, these cells strongly expressed antigens of activated or mature DCs, such as CD40, CD208/DC-LAMP, CCR7, and CD83. Interestingly, all of the double-positive immunostaining results showed that the AIRE^+^ cells exhibited obvious dendritic morphology. Overall, these data indicated that lymph node AIRE^+^ cells represented one subset of interdigitating DCs with the mature/activation phenotype. The above evidence indicated that AIRE-expressing cells in human peripheral tissues were mainly a type of DC subset.

In summary, AIRE is thought to be mainly expressed by mTECs in the thymus and by one type of DC subset in the peripheral lymphoid tissues.

AIRE in cells mainly shows a scattered, speckled distribution in the cell nuclei, which conforms to the transcriptional regulatory function of AIRE in cells. Therefore, AIRE may directly regulate gene expression in the thymus. Endogenous AIRE is only observed in the puncta pattern and it co-localizes with CBP [17, 18]. In transfected cells, AIRE has two expression patterns: a punctate nuclear pattern along the cytoskeleton and a fibrous staining pattern [7, 9, 18]; AIRE mutants in patients were transfected into COS-1 cells and the results showed that mutations of AIRE are associated with changes in these staining patterns [19, 20]. The distribution of AIRE expression suggests that this protein plays a role in the induction and maintenance of immune tolerance that cannot be ignored [21].

## 3. The Function of AIRE in Peripheral Immune Tolerance

### 3.1. Eliminating Auto-Reactive T Cells through Regulation of TSA Expression

Similar to the function in thymic mTECs, AIRE transcriptionally regulates the expression of various TSAs in peripheral lymphoid organs. Gardner et al. studied extra-thymic AIRE-expressing cells (eTACs) in the AIRE-driven Igrp-GFP (Adig) mouse model and detected IGRP expression driven by AIRE in AIRE^+^ mTECs and eTACs in the Adig mice [22]. When islet-specific glucose-6 phosphatase-related protein- (IGRP-) specific CD8^+^ T cells were transferred into the Adig mice, eTAC directly delivered IGRP to these T cells to stimulate the proliferation and death of IGRP-specific CD8^+^ T cells. Therefore, similar to AIRE-expressing mTECs, eTACs can induce tolerance through the clearance of auto-reactive CD8^+^ T cells. In addition, eTACs express different tissue-specific antigen sets than AIRE-expressing mTECs do. AIRE regulates 163 genes in eTACs and 1835 genes in mTECs; however, only 7 overlapping genes are regulated by both peripheral and thymic AIRE. Through the peripheral expression of different TSA groups, eTACs can lead to the tolerance of central antigen-specific T cells that have not been cleared [22]. Some studies have also shown that unlike AIRE-expressing mTECs, eTACs can receive cues from and/or play an important role in local immune responses [23, 24].

Some studies have shown that AIRE expression in DCs does not seem to influence the expression of TSAs, because there were minimal differences in the gene-expression profiles of AIRE-WT and AIRE-KO mice [22, 25, 26]. However, other studies have confirmed that AIRE-expressing DCs in splenocytes promote the expression of the type I diabetes mellitus- (T1D-) associated antigen insulin II (Ins2) [25, 27]. In addition, studies from our group showed that AIRE significantly up-regulated the mRNA expression levels of Ins2, glutamate decarboxylase 65/67 (GAD65/67), insulinoma-associated protein 2 (IA-2), IGRP, retinol-binding protein 3 (Rbp3), salivary protein 1 (Spt1), pyrin domain containing 5 (Nalp5), and mylin-oligodendrocyte glycoprotein (Mog) in AIRE-overexpressing bone marrow-derived dendritic cells (BMDCs), DC2.4 cells, and RAW264.7 cells. In AIRE^−/−^ BMDCs, the expression levels of Ins2, GAD65/67, IA-2, IGRP, Rbp3, Spt1, Nalp5, Mog, and major urinary protein-1 (MUP1) were significantly lower than those in the wild-type- (WT-) BMDC group; in addition, desmoglein 1 alpha (Dsg1a) and ladinin 1 (Lad1) were not expressed in the AIRE^−/−^ BMDCs. These results indicated that AIRE up-regulated the expression of various TSAs in the dendritic cell line DC2.4, the macrophage cell line RAW264.7, and primary bone marrow DCs, whereas the expression of various TSAs was absent in BMDCs from AIRE^−/−^ mice [21, 28, 29].

In summary, the ectopic expression of AIRE in eTACs and APCs can up-regulate the expression of various TSAs, which is similar to the central function. These results suggest that AIRE may regulate TSA expression in eTACs and APCs to clear peripheral auto-reactive T cells and participate in immune tolerance.

### 3.2. Influencing the Antigen-Presenting Ability of APCs by Regulating TLRs and MHC II

Regulation of Toll-like receptors (TLRs), MHC II molecules, and CD86 expression in APCs by AIRE may influence the signals of the interaction between APCs and T cells to affect the antigen-presenting function of the APCs [8].

TLRs are an important type of PRR that are mainly expressed on APCs and can selectively recognise components of pathogenic microorganisms. Currently, 11 human TLRs and 13 mouse TLRs have been identified that can recognise different antigenic epitopes of different pathogenic microorganisms as well as senescent and sick cells [30]. TLRs have two main functions. First, they recognise different pathogens and promote innate and specific immune responses to exert the immune defence function. Second, they promote immune homeostasis via the recognition and clearance of senescent and sick cells [31].

**Figure 1:**
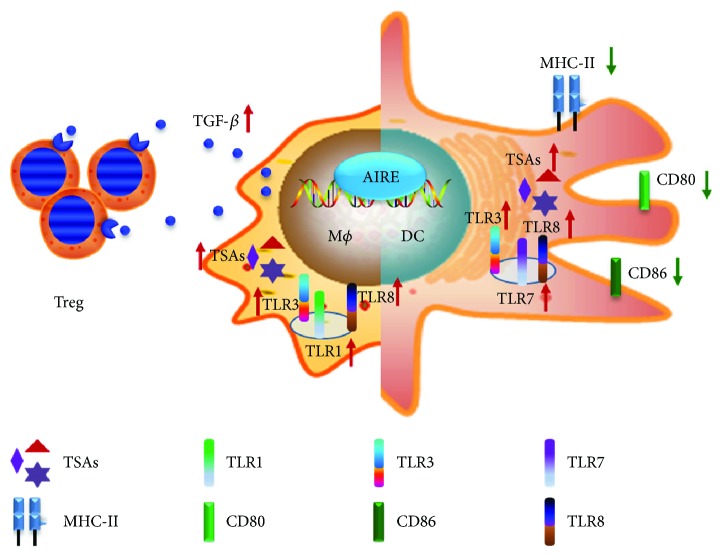
The role of AIRE in macrophage and dendritic cells *in vitro.*

Based on these TLR functions, we speculated that AIRE expressed in APCs might influence TLR expression to further affect the recognition of pathogenic microorganisms, senescent cells, or sick cells by TLRs. Therefore, our previous studies evaluated TLR expression (Figure 1). By establishing a stable AIRE-expressing cell model in the mouse macrophage cell line RAW264.7, the expression of TLRs in cells was first detected. The results showed that in the stable AIRE-expressing RAW264.7 cells, the expression of TLR1, TLR3, and TLR8 increased, especially that of TLR8. In addition, the mRNA expression levels of TLR1, TLR3, and TLR8 increased in mouse peritoneal macrophages with transient AIRE expression. Our results suggested that AIRE might influence macrophages to recognise the nucleic acids of pathogens, senescent cells, or sick cells by regulating TLR expression in macrophages [31]. Next, our group performed in-depth studies of the mechanisms underlying the regulation of TLR expression by AIRE. After DNA-PKcs silencing, TLR1, TLR3, and TLR8 expression was down-regulated in macrophages transiently transfected with pEGFPC1/AIRE. The luciferase reporter gene assay results showed that the transcriptional activities of the TLR1, TLR3, and TLR8 promoters were decreased in the GFP/AIRE RAW cells after DNA-PKcs silencing [32]. These results showed that AIRE could collaborate with DNA-PKcs to up-regulate the expression of TLR1, TLR3, and TLR8 in macrophages. These studies suggested that DNA-PKcs and AIRE interacted to regulate TLR expression. In addition, stimulation by the TLR1 and TLR3 ligands increased the expression of the corresponding downstream target gene products to change the recognition ability of macrophages for pathogens during antigen presentation [31] and played important roles in the maintenance of peripheral immune tolerance.

In addition to macrophages, some reports showed that the antigen-presenting function of DCs alerted. One study showed that the antigen-presenting ability of DCs from APS-1 patients changed compared with that in the control group [11, 25]. Some reports also showed that the antigen-presenting ability of DCs from AIRE^−/−^ mice changed [33]. However, other researchers could not confirm this conclusion [34]. Sun et al. reported that TLR3, TLR7, and TLR8 were up-regulated in AIRE-overexpressing DC2.4 cells [35]. The study of AIRE^+/+^ DCs and AIRE^−/−^ mouse DCs also showed that AIRE maintained the low expression of co-stimulatory molecules and MHC II molecules in BMDCs to exert its immune tolerance function [29].

Based on the results described in the above two paragraphs, it can be inferred from studies on macrophages and dendritic cells that AIRE can regulate the expression of TLRs and surface molecules in APCs. From a functional perspective, AIRE will influence the antigen-presenting ability of APCs and play an important role in peripheral immune responses. In addition, since most of the above are *in vitro* experiments, further studies are needed to confirm this hypothesis.

### 3.3. Mediating DC-Activated T Cells by Influencing the Maturation Status of DCs

DCs can be divided according to their maturation status into mature and immature types. These two types have different cell phenotypes. In addition, DCs at different maturation states can have different effects on T cells. Mature DCs induce effective immune responses via the expression of co-stimulatory molecules and high levels of MHC II molecules, whereas immature DCs usually exert immune suppressive functions because they lack co-stimulatory molecules and have low levels of MHC II molecule expression.

Our study showed that AIRE down-regulated MHC II molecules and the co-stimulatory molecule CD86 to reduce the antigen-presenting ability of APCs, influence cell activation, and maintain the stable status of peripheral immune tolerance. In contrast, when AIRE expression is defective or abnormal, its inhibitory function on MHC II and CD86 may be reduced to boost the function of APCs and activate T cells, resulting in an enhancement of immune responses and the development of autoimmune diseases. The study by our group on the expression of co-stimulatory and MHC II molecules in BMDCs showed that the expression of CD40, CD86, and MHCII molecules was significantly lower in AIRE-BMDCs than it was in GFP-BMDCs [28] and that the expression of CD40, CD80, CD86, and MHCII molecules was significantly higher in the AIRE^−/−^ BMDCs than it was in the WT BMDCs [29] (Figure 1). These results indicated that AIRE maintained the low expression levels of co-stimulatory molecules in BMDCs to exert its immune tolerance function. Another study showed that the enhanced ability of peripheral DCs to activate T cells and increase T cell proliferation in AIRE^−/−^ mice might be caused by the abnormal expression of the co-stimulatory molecule VCAM-1 in AIRE^−/−^ mice [33].

In summary, AIRE can affect DCs by regulating their maturation status to induce the conditions required for T cell activation, regulate the activation status of T cells, and maintain peripheral immune tolerance.

### 3.4. Regulating Immune Responses by Inducing T Cell Tolerance and Differentiation

In central tolerance, AIRE-expressing mTECs promote the clearance or inactivation of auto-reactive T cells through two possible pathways. The first occurs through negative selection, in which AIRE enhances the clonal deletion of auto-reactive thymocytes. The second is mediated by positive selection, in which AIRE promotes the clonal induction of the regulatory T cell (Treg) phenotype in thymocytes [34, 36]. This condition is also present in peripheral tolerance.

Our group co-cultured streptozotocin-induced type 1 diabetes (STZ-T1D) or WT mouse-derived splenocytes with either control or AIRE^+/+^ DCs for 48 h. Apoptosis was detected using flow cytometry. The AIRE-overexpressing DCs induced apoptosis of auto-reactive CD4^+^ T cells to induce their tolerance. A similar method was used for co-culture for 48 h, followed by insulin stimulation for 24 h. The number of IFN^+^ T cells among the CD4^+^ T cells was detected using flow cytometry. The AIRE-transfected DCs induced the functional inactivation of CD4^+^ T cells [3, 28]. In summary, AIRE-overexpressing DCs maintain CD4^+^ T cell tolerance by inducing both the functional inactivation of these cells and the apoptosis of auto-reactive CD4^+^ T cells.

Gardner et al. [22] discovered peripheral-tissue antigen (PTA) transcripts in stromal cells in the lymph nodes and spleen. At least some of these cells expressed the AIRE protein. Transgenes targeting antigens of peripheral stromal cells can induce persistent contact with T cells, induce their complete activation, and then cause their death, indicating that stromal cells in peripheral lymphoid organs can directly present transgene-encoded antigens to T cells to cause the death of activation-induced cells [25].

AIRE in DCs can also influence the differentiation of CD4^+^ T cell subsets by regulating cytokine secretion to regulate immune responses and self-tolerance. Studies by our group on AIRE-overexpressing BMDCs showed that AIRE might induce Treg and Th2 differentiation by promoting transforming growth factor *β* (TGF-*β*) and IL-4 production in BMDCs and decreasing Th1, Th17, Th22, and T_FH_ differentiation through the inhibition of IL-12, TNF-*α*, and IL-6 production, which induces immune tolerance [28].

Among the CD4^+^ T cell subsets, Tregs play critical roles in the induction of tolerance. To investigate the functions of Tregs, one study performed double-thymus transplant and splenocyte transfer experiments. The results indicated that AIRE^−/−^ mice did not develop multiple autoimmune diseases due to the lack of Treg activity [32]. Our previous study also showed that AIRE-overexpressing RAW264.7 macrophage cells co-cultured with splenocytes increased the percentage of CD4^+^ Foxp3^+^ Treg cells. This promotion function directly affected the percentage of CD4^+^ Foxp3^+^ Treg cells through cell–cell contact [37].

Furthermore, as a novel T cell subtype, T_FH_ participates in humoral immunity and exerts its function mainly by regulating the composition of germinal centres and cell responses in germinal centres. Some studies have shown that DCs provide a combination of co-stimulatory signals (inducible co-stimulators (ICOSs)), induction of transcription factor BCL-6 expression, and cytokine-mediated signals (IL-6) to guide T_FH_ cell development [38], which can be further maintained through the autocrine IL-21 pathway [39]. The study of Evelina et al. on AIRE^−/−^ mice showed that up-regulation of ICOS ligand (ICOSL) expression in 33D1^+^ DCs specifically promoted T_FH_ proliferation compared with proliferation in the WT mice. The excessive activation of B cells preserved in splenic follicles resulted from the reduction of CXCL12 secretion by AIRE^−/−^ 33D1^+^ DCs, which also directly mediated T_FH_ proliferation [40].

In summary, similar to central tolerance, peripheral DCs that express AIRE can regulate peripheral tolerance through two possible routes. First, they can promote the apoptosis of auto-reactive CD4^+^ T cells to maintain the tolerance status. Second, they can induce the differentiation of CD4^+^ T cells to mediate T cell responses and induce immune tolerance.

## 4. Conclusion and Outlook

The peripheral functions of AIRE may even be more diverse than its central functions are. First, the types of peripheral tissues and cells with AIRE distributions are more extensive, and the corresponding functions are more diverse. Next, in addition to regulating TSA expression to induce the apoptosis of auto-reactive T cells and the production of Tregs, peripheral AIRE can induce peripheral tolerance by regulating TLR expression in APCs and the maturation status of APCs. The potential diverse functions of peripheral AIRE enrich AIRE-related studies and provide more targets as entry points for treating autoimmune diseases using AIRE. However, most of the above peripheral functions of AIRE are obtained from *in vitro* experiments, for the clear roles of AIRE in the periphery still require more studies *in vivo* as well as other evidences to support them. In addition, the specific mechanisms of AIRE in the periphery and whether AIRE has other functions in peripheral tolerance are not clear; therefore, more relevant research is needed in this area.

## References

[1] Anderson M. S., Su M. A. (2016). AIRE expands: new roles in immune tolerance and beyond. *Nature Reviews Immunology*.

[2] Bruserud Ø., Oftedal B. E., Wolff A. B., Husebye E. S. (2016). AIRE-mutations and autoimmune disease. *Current Opinion in Immunology*.

[3] Adamson K. A., Pearce S., Lamb J. R., Seckl J. R., Howie S. E. M. (2004). A comparative study of mRNA and protein expression of the autoimmune regulator gene (Aire) in embryonic and adult murine tissues. *Journal of Pathology*.

[4] Perniola R. (2018). Twenty years of AIRE. *Frontiers in Immunology*.

[5] Eldershaw S. A., Sansom D. M., Narendran P. (2011). Expression and function of the autoimmune regulator (Aire) gene in non-thymic tissue. *Clinical and Experimental Immunology*.

[6] Kawano H., Nishijima H., Morimoto J. (2015). Aire expression is inherent to most medullary thymic epithelial cells during their differentiation program. *Journal of Immunology*.

[7] Heino M., Peterson P., Sillanpää N. (2000). RNA and protein expression of the murine autoimmune regulator gene (Aire) in normal, RelB-deficient and in NOD mouse. *European Journal of Immunology*.

[8] Yang W. (2008). *The Effect of Autoimmune Regulator (AIRE) on the Functions of APC in Peripheral Immune Tolerance*.

[9] Halonen M., Pelto–Huikko M., Eskelin P., Peltonen L., Ulmanen I., Kolmer M. (2001). Subcellular location and expression pattern of autoimmune regulator (Aire), the mouse orthologue for human gene defective in autoimmune polyendocrinopathy candidiasis ectodermal dystrophy (APECED). *Journal of Histochemistry & Cytochemistry*.

[10] Poliani P. L., Kisand K., Marrella V. (2010). Human peripheral lymphoid tissues contain autoimmune regulator-expressing dendritic cells. *American Journal of Pathology*.

[11] Pöntynen N., Strengell M., Sillanpää N. (2008). Critical immunological pathways are downregulated in APECED patient dendritic cells. *Journal of Molecular Medicine*.

[12] Heino M., Peterson P., Kudoh J. (1999). Autoimmune regulator is expressed in the cells regulating immune tolerance in thymus medulla. *Biochemical and Biophysical Research Communications*.

[13] Suzuki E., Kobayashi Y., Kawano O. (2009). Expression of AIRE in thymocytes and peripheral lymphocytes. *Autoimmunity*.

[14] Perniola R. (2012). Expression of the autoimmune regulator gene and its relevance to the mechanisms of central and peripheral tolerance. *Clinical & Developmental Immunology*.

[15] Mellor A. L., Keskin D. B., Johnson T., Chandler P., Munn D. H. (2002). Cells expressing indoleamine 2,3-dioxygenase inhibit T cell responses. *Journal of Immunology*.

[16] Zheng X., Yin L., Liu Y., Zheng P. (2004). Expression of tissue-specific autoantigens in the hematopoietic cells leads to activation-induced cell death of autoreactive T cells in the secondary lymphoid organs. *European Journal of Immunology*.

[17] Ferguson B. J., Alexander C., Rossi S. W. (2008). AIRE’s CARD revealed, a new structure for central tolerance provokes transcriptional plasticity. *Journal of Biological Chemistry*.

[18] Su M. A., Anderson M. S. (2004). Aire: an update. *Current Opinion in Immunology*.

[19] Halonen M., Kangas H., Rüppell T. (2004). APECED-causing mutations in AIRE reveal the functional domains of the protein. *Human Mutation*.

[20] Ramsey C., Bukrinsky A., Peltonen L. (2002). Systematic mutagenesis of the functional domains of AIRE reveals their role in intracellular targeting. *Human Molecular Genetics*.

[21] Zhu W., Chen X., Liao X., Hu Z., Huang W., Zeng Z. (2017). Autoimmune regulator affects macrophage polarization in murine RAW 264.7 cells. *International Journal of Clinical and Experimental Medicine*.

[22] Gardner J. M., DeVoss J. J., Friedman R. S. (2008). Deletional tolerance mediated by extrathymic Aire-expressing cells. *Science*.

[23] Lee J. W., Epardaud M., Sun J. (2007). Peripheral antigen display by lymph node stroma promotes T cell tolerance to intestinal self. *Nature Immunology*.

[24] Fletcher A. L., Lukacs-Kornek V., Reynoso E. D. (2010). Lymph node fibroblastic reticular cells directly present peripheral tissue antigen under steady-state and inflammatory conditions. *Journal of Experimental Medicine*.

[25] Mathis D., Benoist C. (2009). Aire. *Annual Review of Immunology*.

[26] Kont V., Laan M., Kisand K., Merits A., Scott H. S., Peterson P. (2008). Modulation of Aire regulates the expression of tissue-restricted antigens. *Molecular Immunology*.

[27] Oliveira E. H., Macedo C., Donate P. B. (2013). Expression profile of peripheral tissue antigen genes in medullary thymic epithelial cells (mTECs) is dependent on mRNA levels of autoimmune regulator (Aire). *Immunobiology*.

[28] Li D., Zhao B., Luo Y. (2017). Transplantation of Aire-overexpressing bone marrow-derived dendritic cells delays the onset of type 1 diabetes. *International Immunopharmacology*.

[29] Huo F., Li D., Zhao B. (2018). Deficiency of autoimmune regulator impairs the immune tolerance effect of bone marrow-derived dendritic cells in mice. *Autoimmunity*.

[30] Wei T., Gong J., Rössle S. C., Jamitzky F., Heckl W. M., Stark R. W. (2011). A leucine-rich repeat assembly approach for homology modeling of the human TLR5-10 and mouse TLR11-13 ectodomains. *Journal of Molecular Modeling*.

[31] Zhu W., Yang W., He Z. (2011). Overexpressing autoimmune regulator regulates the expression of toll-like receptors by interacting with their promoters in RAW264.7 cells. *Cellular Immunology*.

[32] Wu J., Zhu W., Fu H. (2012). DNA-PKcs interacts with Aire and regulates the expression of toll-like receptors in RAW264.7 cells. *Scandinavian Journal of Immunology*.

[33] Ramsey C., Hässler S., Marits P. (2006). Increased antigen presenting cell-mediated T cell activation in mice and patients without the autoimmune regulator. *European Journal of Immunology*.

[34] Anderson M. S., Venanzi E. S., Chen Z., Berzins S. P., Benoist C., Mathis D. (2005). The cellular mechanism of Aire control of T cell tolerance. *Immunity*.

[35] Sun J., Niu K., Fu H., Li H., Li Y., Yang W. (2016). Autoimmune regulator expression in DC2.4 cells regulates the NF-*κ*B signaling and cytokine expression of the Toll-like receptor 3 pathway. *International Journal of Molecular Sciences*.

[36] Yang S., Fujikado N., Kolodin D., Benoist C., Mathis D. (2015). Regulatory T cells generated early in life play a distinct role in maintaining self-tolerance. *Science*.

[37] Sun J., Fu H., Wu J., Zhu W., Li Y., Yang W. (2013). Macrophages overexpressing Aire induce CD4^+^Foxp 3^+^ T cells. *Molecular Medicine Reports*.

[38] Choi Y. S., Kageyama R., Eto D. (2011). ICOS receptor instructs T follicular helper cell versus effector cell differentiation via induction of the transcriptional repressor Bcl 6. *Immunity*.

[39] Qi H. (2016). T follicular helper cells in space-time. *Nature Reviews Immunology*.

[40] Lindmark E., Chen Y., Georgoudaki A.-M. (2013). AIRE expressing marginal zone dendritic cells balances adaptive immunity and T-follicular helper cell recruitment. *Journal of Autoimmunity*.

